# CO_2_ geological storage site selection and long-term potential assessment framework based on TOUGH2/ECO2N

**DOI:** 10.1371/journal.pone.0321715

**Published:** 2025-04-24

**Authors:** Wenjie Sun, Wen Liu, Shunli Ren, Gang Lin, Chenxu Wang, Xiaodong Jiang

**Affiliations:** 1 College of Geoscience and Surveying Engineering, China University of Mining & Technology-Beijing, Beijing, China; 2 Shenhua Geology Exploration Co., Ltd, Beijing, China; 3 Institute of Geographic Sciences and Natural Resources Research, Chinese Academy of Sciences, Beijing, China; Independent Researcher, Global Talent Exceptional Promise, UNITED KINGDOM OF GREAT BRITAIN AND NORTHERN IRELAND

## Abstract

Carbon dioxide capture and storage (CCS) technology is considered a crucial tactic for achieving the “dual carbon” goals. However, current CCS technologies face several challenges, such as the absence of a comprehensive and systematic set of criteria for site selection, coupled with the lack of a universally applicable evaluation framework.To address these issues, this study proposes a regional-scale CO_2_ geological storage suitability assessment and potential estimation framework that can be adapted to similar regions. Using the Yellow River Delta as a case study, a geological storage site selection indicator system was developed, and a model for assessing site suitability was constructed.The model was employed to evaluate the appropriateness of CO_2_ geological sequestration sites within the study region, and numerical simulations of CO_2_ storage were performed utilizing the TOUGH2/ECO2N simulator for prospective target zones. This research examines the migration dynamics of CO_2_ in saline aquifers, the progression of storage mechanisms, and the carbon sequestration capacity of various storage configurations. The research addresses key questions such as: How can the optimal CO_2_ geological storage target area be selected? How can the best storage strategy matching the target area be determined? How can the migration and distribution characteristics of CO_2_ after storage be simulated? Are there potential risks in the future?

## Introduction

With the accelerated advancement of global industrialization and escalating energy demands, anthropogenic carbon dioxide (CO_2_) emissions have exhibited a sustained upward trajectory, constituting a principal driver of contemporary climate change [1]. Achieving net-zero CO2 emissions by mid-century and limiting global temperature rise to 2°C is crucial for sustainable development and a shared global challenge [2]. The strategies recommended by COP 21 involve boosting energy conservation and efficiency, using renewable and low - carbon fuels, implementing geo - engineering like afforestation, and, most importantly, developing CO2 capture and storage (CCS) techniques [3]. CCS has thus become a key strategy for pursuing sustainable development and combating global warming [4,5].

The choice of geological storage medium is critical to the effectiveness of CO_2_ geological sequestration. Common storage media include deep saline aquifers, depleted petroleum and natural gas reservoirs, unextractable coal seams, and basaltic formations.Among these, deep saline aquifers are regarded as among the most efficient and viable options for carbon dioxide sequestration [6]. These saline aquifers are located deep underground, where the groundwater is highly mineralized and unsuitable for agricultural or drinking purposes. Such geological structures are more common than oil and gas fields, and they offer greater sequestration potential. A portion of the injected CO_2_ will undergo dissolution in the aqueous phase, and throughout its migration within the reservoir,CO_2_ will react with the saline water and surrounding rock, enhancing its storage capacity [7].

The evaluation of CO_2_ geological sequestration potential mainly relies on experimental conditions, geological characteristics, and theoretical calculations, but there is still no unified standard or methodology for assessment [8]. Researchers typically develop relevant evaluation criteria and weightings based on the geological characteristics of the storage site and the specific evaluation objectives. In the early evaluation stages, initial screening is based on factors such as reservoir characteristics, fluid properties, and surface infrastructure. An appropriate suitability index system is then designed based on geological characteristics and evaluation goals [9]. After the initial screening, further detailed evaluation and ranking of potential storage reservoirs are carried out to establish a basin-level evaluation index system. One of the most representative examples of this system is the 15-indicator framework proposed by Bachu [10], which he used to assess the sequestration potential of the Canadian Basin. Over time, more comprehensive evaluation systems have been developed, incorporating considerations of regional geology, local protection, social health, and sequestration safety, such as the system developed by Oldenburg et al [11,12], which builds on Bachu’ framework and adds aspects of health, safety, and environmental risk.

To date, several challenges remain in the CO_2_ geological sequestration site evaluation methods and selection criteria. First, the assessment of sequestration capacity and injection rates requires a detailed study of the physical and chemical properties of the storage medium [6,13]. Second, existing site evaluation criteria are not comprehensive, lacking a widely applicable evaluation system with a high degree of quantifiability. Some key factors, such as fault development and sequestration safety, are still evaluated qualitatively or based on subjective judgment, which calls for further improvement in the scientific rigor of the evaluation process. Moreover, sequestration safety remains a concern, particularly regarding potential leakage risks and seismic events triggered by CO_2_ injection [14]. Additionally, the interaction between CO_2_ and the storage medium can alter the physical and chemical properties of the medium, potentially affecting the sequestration outcome [15]. Moreover, the widespread implementation of Carbon Capture, Utilization, and Storage (CCUS) is contingent not only upon technological advancements and energy demands but also on societal acceptance [16–18].The reasons hindering the development of CCS technology are not only technical but also related to cost issues [19–22].As, despite relying on existing and developing components, large-scale implementation of CCS faces financial barriers and challenges in deploying extensive projects [23].For example, in a region with multiple carbon emission sources and storage sinks, the properties of these sources and sinks vary, with each source having a different carbon emission level and each sink having a different sequestration capacity. The costs of capturing or storing CO_2_ also vary. Therefore, how to select and match suitable sources and sinks to minimize the total cost for a given carbon reduction target is a key research issue—the source-sink matching problem. Current CCS deployments have not fully considered the entire carbon capture chain, overlooking spatial matching between CO_2_ sources and sinks and the post-sequestration migration patterns and their impacts [24].

This study focuses on the Yellow River Delta and proposes a comprehensive CO_2_ geological sequestration site selection and potential assessment framework. Based on this framework, a comprehensive CO_2_ geological sequestration site selection and suitability evaluation index system is developed, which integrates the principles and mechanisms of CO_2_ sequestration in saline aquifers. The system takes into account regional geological background, social environment, and economic suitability conditions, scientifically defining the spatial distribution types and ranges of CO_2_ geological sequestration sites, as well as determining the priority zones and optimal storage target areas. On this basis, a multi-objective decision-making CO_2_ geological sequestration source-sink matching model is established to identify the appropriate emission sources for each target area. Finally, a CO_2_ geological sequestration numerical model is developed for all target areas, considering both gaseous and liquid phases of CO_2_. The model simulates the migration process and migration patterns of CO_2_ during sequestration and determines the best sequestration plans. This study addresses the following key questions: How should the optimal CO_2_ geological sequestration target area be selected? How can the best sequestration plan for the target area be determined? How can the post-sequestration CO_2_ migration distribution characteristics be simulated? And are there potential risks in the future? The research provides a framework and methodology for regional-scale CO_2_ geological sequestration suitability evaluation and potential estimation, which can be reasonably adapted to other similar regions.

## Methods

**Fig 1:**
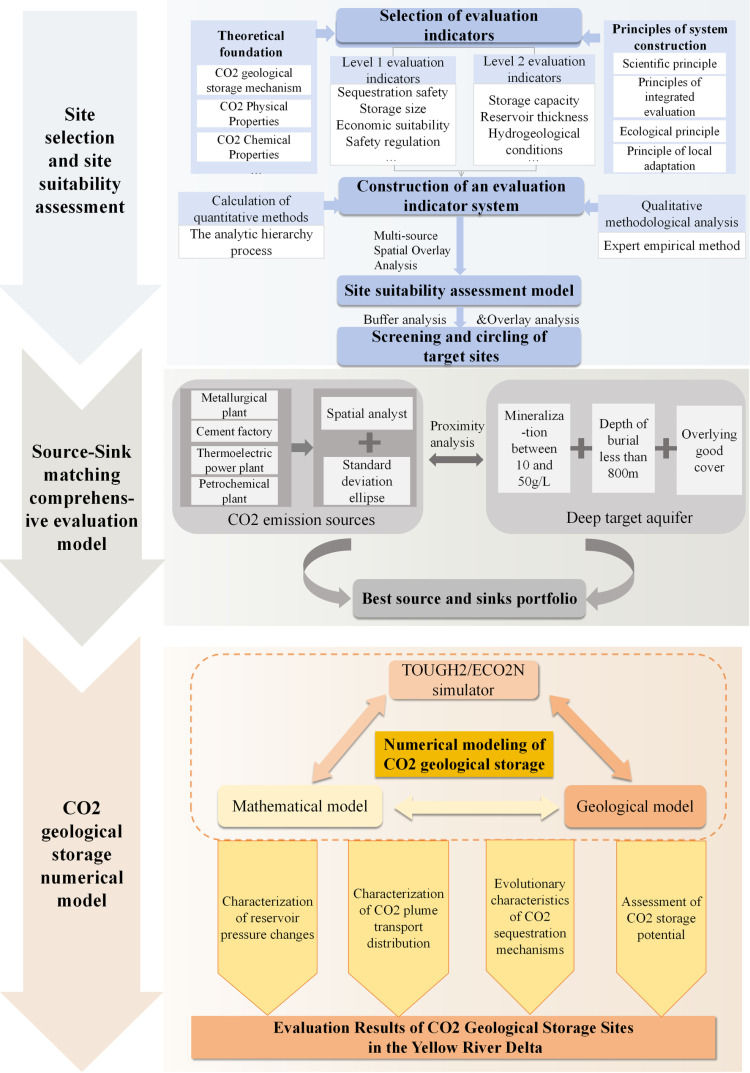
Technology Roadmap.

This study focuses on the Yellow River Delta and proposes a comprehensive framework for CO_2_ geological storage site selection and potential evaluation. The Yellow River Delta is located in Shandong Province, where energy, electricity, and chemical industries with high carbon emissions are widely distributed. According to the “Survey and Evaluation of Geological Carbon Sink and Carbon Storage Resources in Key Regions” project by the China Geological Survey, the goal is to identify CO_2_ geological storage sites in the region with the capacity to store millions of tons of CO_2_ per year, in order to support the development of industrial-scale CO_2_ geological storage plans for the high-carbon emission zones along the eastern coastal area. This will contribute to the optimization and adjustment of the regional energy structure.The technical roadmap is shown in Fig 1.

### CO_2_ geological storage site selection and site suitability assessment

The construction of evaluation indicators follows the principles of scientific rigor, comprehensive assessment, ecological sustainability, and adaptability to local conditions. The evaluation is based on five key indicators: storage safety, storage capacity, economic feasibility, social and environmental stability, and safety supervision. Taking into account the mechanisms of saline aquifer CO_2_ storage, geological characteristics of potential storage sites, environmental risks, and the relevant properties of CO_2_, a CO_2_ geological storage site evaluation system was established, referring to the domestic and international CO_2_ geological storage site evaluation frameworks [25–29]. The Analytical Hierarchy Process (AHP) was applied to calculate the weights of the evaluation indicators, with a consistency ratio (CR) value of less than 0.1, indicating that the consistency test was passed.

In the process of classifying site suitability, a multi-objective comprehensive assessment method was employed, integrating both qualitative and quantitative factors based on GIS spatial analysis tools. Through the spatial overlay of multi-source information, a suitability classification of CO_2_ saline aquifer storage sites and target area selection was carried out, and a corresponding suitability assessment model was constructed, identifying suitable sites. Further qualitative analysis was conducted in combination with the evaluation indicators system and relevant geological data and literature. Finally, the suitability classification of CO_2_ geological storage sites in the Yellow River Delta and the geological storage target area were determined.

### Source-sink matching comprehensive evaluation model

Spatial analyst and Standard deviation ellipse tools were employed to analyze the distribution characteristics of 198 CO_2_ emission sources in the Yellow River Delta. The study selected aquifers in semi-closed and closed hydrogeological structures, located at depths of less than 800 meters, with a good cap layer and characterized by slow or very slow hydrological exchange that is not exploitable under current development conditions[30]. To optimize CO_2_ storage capacity, a nearest-neighbor analysis method was employed, utilizing CO_2_ emission source. The deep saline aquifer storage sites were considered as the sinks. With a 100 km search radius, the proximity distribution of CO_2_ emission sources and deep saline aquifers in the Yellow River Delta was matched, revealing the spatial relationships between various data elements.

### CO_2_ geological storage numerical model

The mathematical model for the flow of CO_2_ fluid, based on the physical and chemical properties of supercritical CO_2_ fluid, is constructed with the following assumptions:

(1) The CO_2_ geological storage target saline aquifer is approximated as a continuous porous media model.
(2) The gas phase CO_2_ and the liquid phase saline water, as well as the multiphase flow in the target saline aquifer, all satisfy the generalized Darcy’s law.
(3) All fluids in the deep saline aquifer obey thermal equilibrium, chemical equilibrium, and mechanical equilibrium.
(4) Chemical components other than NaCl and H2O in the target saline aquifer are neglected.
(5) Chemical reaction processes in the formation, except for mineral sequestration, are not considered.
(6) The influence of the formation stress in the target area is temporarily ignored.

The motion of fluid within the pores is described using the generalized Darcy’s law, as represented by the following equation.:


$$q_{\alpha }=\frac{K\cdot K_{\gamma \alpha }}{\mu_{\alpha }}(\nabla P_{\alpha }+\rho_{\alpha }\cdot g\cdot \nabla z)$$ (1)

where:

*μ*_α_is the fluid viscosity (kg/m/s); $\rho_{\alpha }$ is the fluid density (kg/m³); $K_{\gamma \alpha }$ is the relative permeability of the fluid (mD);g is the gravitational acceleration (m/s²), in the downward direction;z is the vertical direction, opposite to the direction of gravity;K is the absolute permeability of the porous medium, which is independent of the direction.

In the numerical simulation of CO_2_ geological storage, the mass and energy conservation principles for fluid flow in the reservoir are used as the basis. The simulation region is discretized using the finite difference method (FDM). Each grid cell satisfies the mass and energy conservation control equations for fluid and heat flow in a multiphase, multi-component system:


$$\frac{d}{dt}\int_{V_{n}}M^{\kappa }dV_{n}=\int_{\Gamma_{n}}F^{\kappa }\cdot nd\Gamma +\int_{V_{n}}q^{\kappa }dV$$ (2)

where:

The integration area $V_{n}$ is any sub-area within the study area corresponding to the dissected grid, m^3^; and the sub-area $\Gamma_{n}$ is the boundary, m^2^; *F* is the mass or heat passing through the unit area, kg or J; *q* is the source-sink term, m3/s; *n* is the normal vector of the surface cell, pointing inward the $V_{n}$; $M^{\kappa }$ is the mass of the component *κ*, kg, whose superscripts *κ* are taken as 1,2,3,4 for water, salt, CO_2_, and heat.

**Fig 2:**
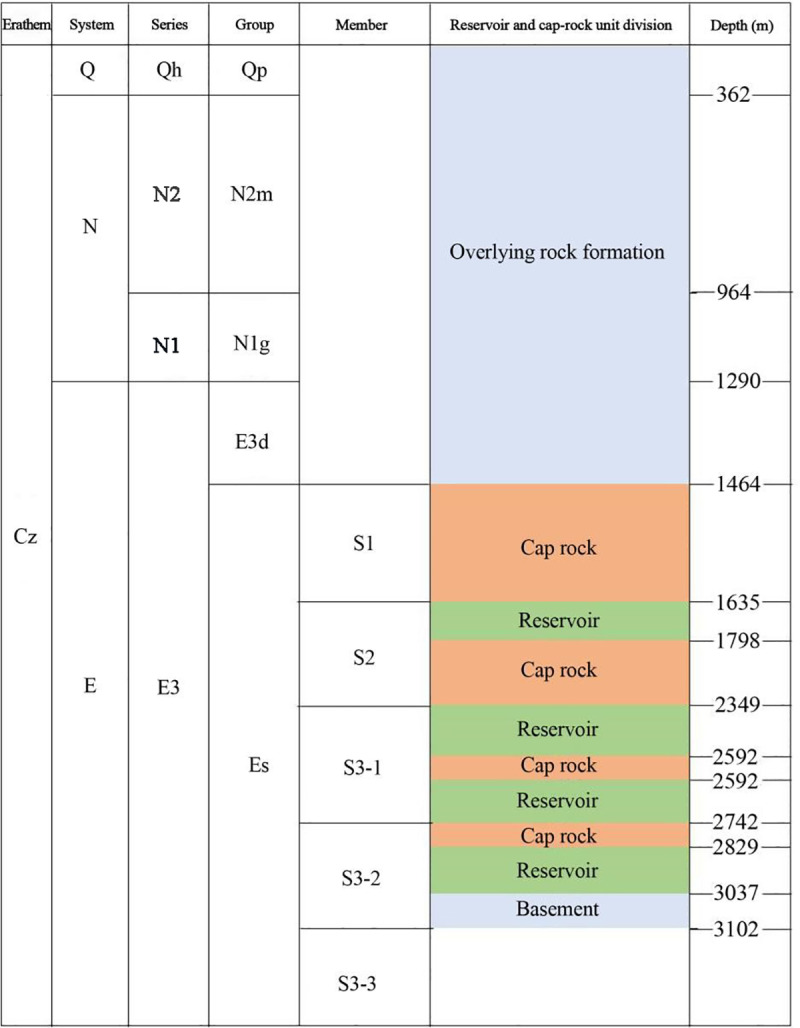
Reservoir and cap unit delineation (Cz:Cenozoic Era;Q:Quaternary Period;N:Neogene Period;E:Paleogene Period;Qh: Holocene Series;N2: Pleistocene Series;N1: Miocene Series;E3: Oligocene Series;Qp: Pingyuan Formation;N2m: Minghuazhen Formation;N1g: Guantao Formation;E3d:Dongying Formation;Es: Shahejie Formation;S1:Member 1 of Shahejie Formation;S2: Member 2 of Shahejie Formation; S3-1: Upper Member 3 of Shahejie Formation;S3-2: Middle Member 3 of Shahejie Formation;S3-3: Lower Member 3 of Shahejie Formation).

The research focuses on the four CO_2_ geological storage caprock combinations of the target area in the Yellow River Delta. Numerical simulation of CO_2_ geological sequestration is carried out using the TOUGH2/ECO2N simulation software. The strata associated with the development of the target zone are shown in Fig 2.The four caprock combinations are distributed across Segments 1, 2, and 3 of the Shahejie Formation. The Shahejie Segment 1 consists of gray mudstone interbedded with bioclastic limestone, dolomite, oil shale, and siltstone; the Shahejie Segment 2 is well developed and consists mainly of mudstone, sandstone, and conglomerate sandstone layers. Segment 3 of the Shahejie Formation is the thickest, dominated by blocky fine sandstone, siltstone, oil shale, mudstone, and shale. A three-dimensional average model is established to analyze the migration and movement characteristics of injected CO_2_ in the reservoir, phase transitions, and CO_2_ storage potential under different sequestration types. The parameters employed in the simulation are presented in Table 1.

**Table 1:** Numerical model simulation parameters(S1:Member 1 of Shahejie Formation;S2: Member 2 of Shahejie Formation;S3-1: Upper Member 3 of Shahejie Formation;S3-2: Middle Member 3 of Shahejie Formation;S3-3: Lower Member 3 of Shahejie Formation).

Parameters	S1 cover layer	S2 reservoir	S2 cover layer	S3-1. upper reservoir	S3-1 center cover.	S3-1 lower reservoir.	S3-2 cover layer.	S3-2 reservoir.
Thickness/*(m)*	171	163	551	243	35	115	87	208
Permeability/*(m*^*2*^)	1×10^-15^	5.2×10^-13^	1×10^-15^	5.5×10^-14^	1×10^-15^	1.1×10^-13^	1×10^-15^	3.8×10^-14^
Porosity	0.1	0.28	0.1	0.27	0.1	0.217	0.1	0.188
Particle density/ $\left(kg\cdot m^{-3}\right)$	2600							
Rock thermal conductivity/ $\left(w\cdot m^{-1}\cdot {�}^{-1}\right)$	2.51							
Enthalpy of rock particles/ $\left(J\cdot kg^{-1}\cdot {�}^{-1}\right)$	920							
Bulk Modulus $/Pa^{-1}$	4.5×10^-10^							
Salinity $/X_{NaCl}/\%$	0.15							
Residual Saturation $S_{rl}$	0.30							
Residual Saturation $S_{gr}$	0.05							
Lambda λ	0.457							

## Results and analysis

### CO_2_ geological storage site selection and site suitability assessment

This study establishes a multi-level hierarchical evaluation index system for CO₂ geological sequestration sites in the Yellow River Delta, integrating sequestration mechanisms in deep saline aquifers, geological settings, environmental risks, and CO₂ physicochemical properties. The system comprises 5 primary indicators and 44 secondary indicators, employing a five-level scoring criteria. Indicator weights were assigned using the Analytic Hierarchy Process (AHP) method (Table 2), and a comprehensive scoring model for evaluation units was developed (Table 3), providing a methodological framework for quantitative assessment of sequestration sites.

**Table 2:** Evaluation indicator weights.

Criteria Layer	Weight	Indicator Layer	Weight
Sealing Safety	0.3894	Lithology of the Main Cap Rock	0.0228
		Continuity of Cap Rock Distribution	0.0266
		Thickness of Cap Rock	0.0262
		Permeability of Cap Rock	0.0216
		Depth of Cap Rock	0.0346
		Sealing Capability of Cap Rock	0.0392
		Secondary Containment Capacity Above the Primary Cap Rock	0.0162
		Peak Ground Acceleration	0.0276
		Number of Historical Earthquakes	0.0213
		Presence of Active Faults	0.0290
		Hydrodynamic Conditions	0.0200
		Hydraulic Head State of Deep Saline Water Layers	0.0220
		Development of Fractures and Faults	0.0276
		Presence of Mining Subsidence, Karst Collapse, or Ground Subsidence Zones in the Area and Surroundings	0.0263
		Presence of Other Wells Deeper than 800m and Abandoned Wells in the Target Area	0.0284
Storage Capacity	0.3795	Thickness of Reservoir	0.0281
		Lithology of the Reservoir	0.0277
		Depth of the Reservoir	0.0431
		Area of the Reservoir	0.0242
		Heterogeneity of the Reservoir	0.0240
		Fluid Mineralization of the Reservoir	0.0241
		Porosity of the Reservoir	0.0219
		Permeability	0.0249
		Presence of Integrity and Sealing Quality in the Cap Rock	0.0291
		Storage Capacity	0.0222
		Geothermal Heat Flow Value	0.0280
		Geothermal Gradient	0.0261
		Surface Temperature	0.0179
		Effective Storage Capacity	0.0186
		Operational Lifespan	0.0197
Economic Suitability	0.0878	Scale of Carbon Source	0.0234
		Transportation Method	0.0155
		Distance from Carbon Emission Sources	0.0140
		Mineral Deposit Conditions	0.0259
Social and Environmental Stability	0.1433	Population Density	0.0235
		Land Use Conditions	0.0188
		Distance to Residential Areas	0.0300
		Public Acceptance	0.0264
		Presence in Protected or Sensitive Areas	0.0219
		Distance to Surface Water Resources	0.0226

**Table 3:** Consistency test results.

Project	Criterion Layer	Sealing Safety	Storage Scale	Economic Feasibility	Social Environmental Stability
CR Value	0.008	0.039	0.011	0.029	0.007
Maximum Eigenvalue	4.021	15.87	15.248	4.076	6.042

**Fig 3:**
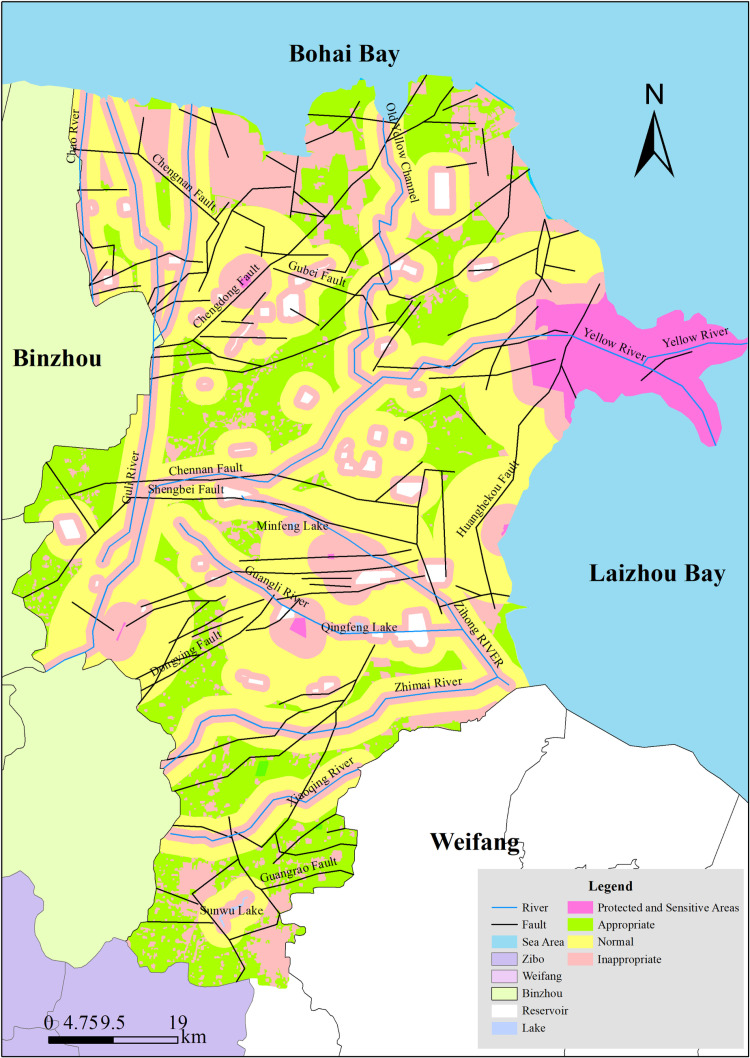
CO2 geological storage site suitability zoning map (Reprinted from http://bzdt.ch.mnr.gov.cn/underaCCBYlicense, with permission from Ministry of Natural Resources, original copyright 2020).

**Fig 4:**
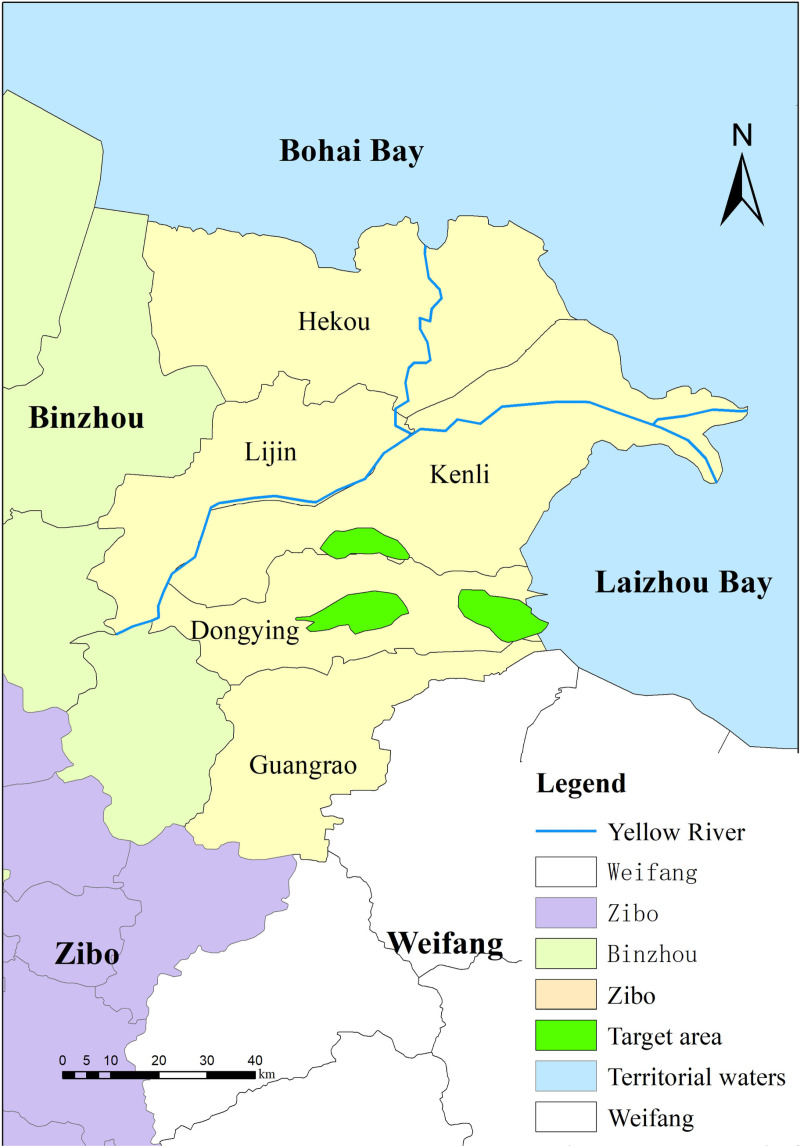
Target area of CO2 geological storage in the study area (Reprinted from http://bzdt.ch.mnr.gov.cn/underaCCBYlicense, with permission from Ministry of Natural Resources, original copyright 2020).

Building upon the CO₂ geological sequestration site evaluation index system, a suitability classification map for sequestration sites was constructed through GIS spatial overlay analysis (Fig 3). This integrated multidimensional constraints including drinking water source areas, ecologically sensitive zones, land use types, spatial proximity to residential areas, fault structures, and reservoir-caprock assemblage characteristics. Subsequently, optimal target sequestration zones were identified and delineated (Fig 4).

### Source-sink matching comprehensive evaluation model

**Fig 5:**
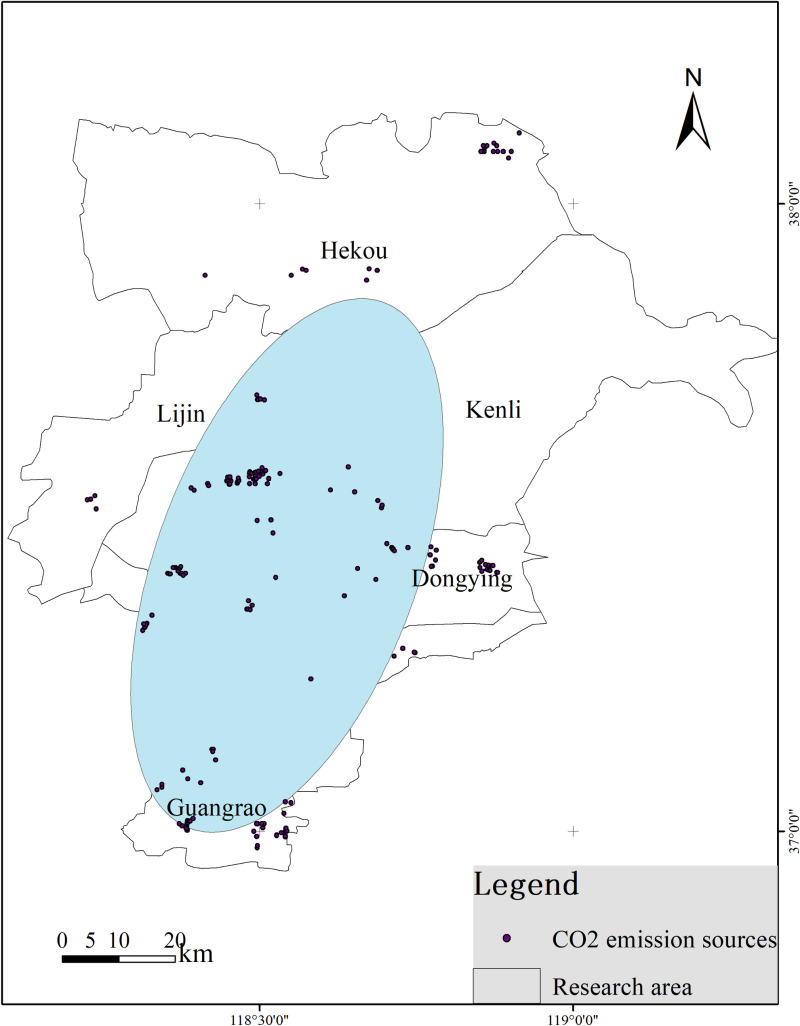
Elliptical analysis of carbon emission sources results (Reprinted from http://bzdt.ch.mnr.gov.cn/underaCCBYlicense, with permission from Ministry of Natural Resources, original copyright 2020).

**Fig 6:**
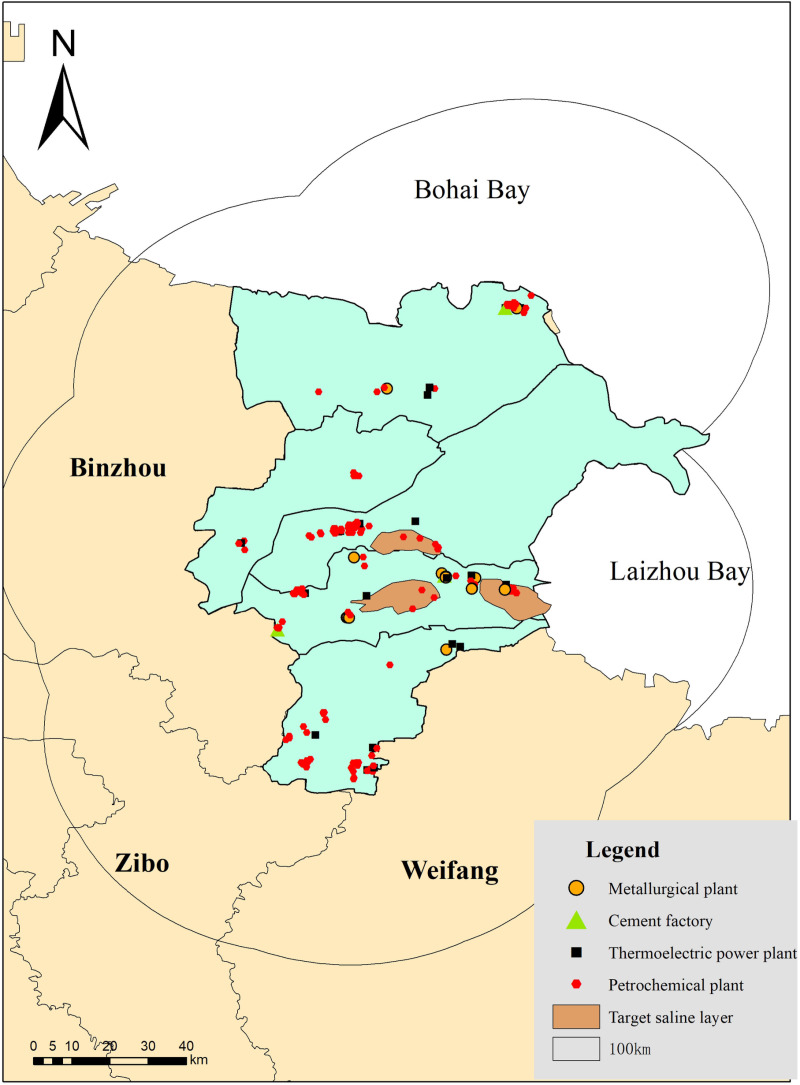
Neighboring analysis results (Reprinted from http://bzdt.ch.mnr.gov.cn/underaCCBYlicense, with permission from Ministry of Natural Resources, original copyright 2020).

A spatial analysis of CO_2_ emission sources in the Yellow River Delta revealed the formation of four high-density clusters, with a clear concentration in the Kenli District and Guangrao County, showing a spatial distribution along a southwest-northeast axis (Fig 5). By analyzing the carbon emissions and distribution of various types of carbon sources across different emission levels, as well as their emission characteristics, the Shahejie Formation was identified as the target saline aquifer for CO_2_ sequestration, based on the principles of CO_2_ saline aquifer sequestration. Using proximity analysis within a 100 km search radius, all CO_2_ emission sources in the Yellow River Delta were successfully matched with suitable sequestration sites, forming source-sink pairs. The total CO_2_ emissions in the study area amounted to 143.58 Mt, with an average CO_2_ emission of 0.73 Mt per source, and transport distances ranging from 0 to 61.94 km (Fig 6).

### CO_2_ geological storage numerical model

The model includes four CO_2_ storage layers, each with ten equidistant injection points, and the CO_2_ injection is performed at 1Mt/a, i.e., 0.7927 kg/s for each injection point, and the total simulation time is 1,000 years with 10 consecutive years of injection.

**Fig 7:**
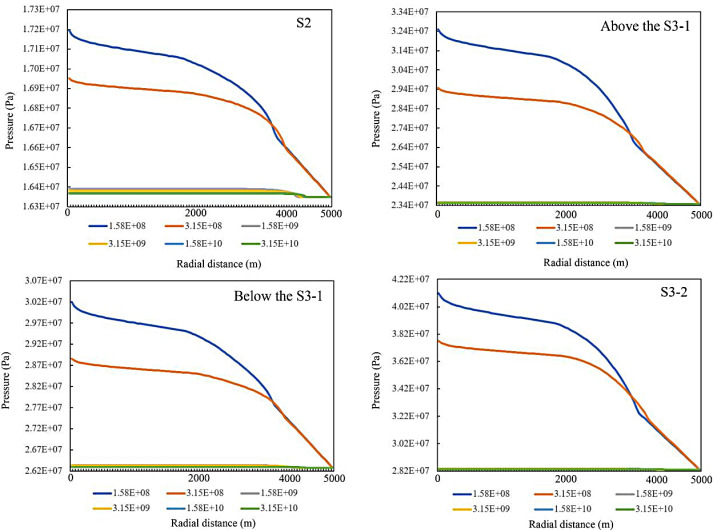
Pressure variation in different strata over time.

To ensure the successful execution of CO_2_ geological storage and the safe confinement of CO_2_ within the deep target saline aquifer, the injection pressure during CO_2_ injection is typically required to be lower than the fracture closure pressure of the formation [31], and it is known that the formation fissure closure pressure is generally the hydrostatic pressure of the formation. As shown in the study of J. Rutqvist et al [32], the fracture closure pressure of the formation is typically 1.5 times the static water pressure of the formation [33]. According to the distribution law of pressure gradient in the target area, the hydrostatic pressures at the lower interface of the four cap layers in the study area are 16.4 MPa, 17.9 MPa, 25.9 MPa and 28.2 MPa, respectively, and the maximum fracture pressure is set to 16.4 MPa, 17.9 MPa, 25.9 MPa and 28.2 MPa, respectively, according to the above model. The fracture pressure calculation model to set the maximum formation bearing pressure, the upper limit of pressure at the top interface of S2 reservoir, S3-1 upper reservoir, S3-1 lower reservoir and S3-2 reservoir are 24.6MPa, 35.3MPa, 39.5MPa, 42.5MPa, respectively.As shown in Fig 7 for the distribution of pressure buildup, this study selects the pressure change of different time periods at the top interface of each storage layer condition. The maximum pressure values of the four groups of storage layers reach 17.2MPa, 32.5MPa, 30.2MPa, and 41.2MPa, respectively,and it can be seen that the peak values of pressure changes caused by the injection pressure in the four groups of brackish water aquifers do not exceed the preset maximum formation.

During injection, the CO_2_ in its gas phase primarily forms a funnel-shaped distribution, with restricted lateral migration at the bottom. As the radial distance from the wellbore increases, the specific gravity (SG) of CO_2_ gradually decreases. The radial flow distance at the base of the caprock is relatively large, causing the SG of CO_2_ at the upper part of the injection well to gradually increase. Under the effects of convection and diffusion, the CO_2_ fluid gradually migrates outward from the vicinity of the wellbore. When the injection is halted, the maximum horizontal migration distances of the CO_2_ plume in the four reservoirs were 1500 m, 1100 m, 1300 m, and 1200 m, in that order.

**Fig 8:**
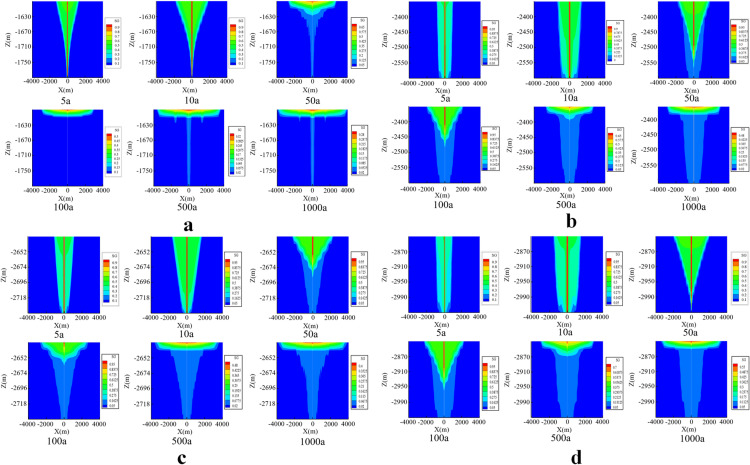
Spatial migration and distribution characteristics of CO2 plume over 0-1000 years in different cap-rock layers (a: S2 Layer; b: Upper S3-1 Layer; c: Lower S3-1 Layer; d: S3-2 Layer).

The high-pressure zone that formed near the wellbore quickly dissipates once CO_2_ injection is completed, and the CO_2_ plume continues to spread laterally as a result of diffusion driven by concentration gradients. During the monitoring phase after CO_2_ injection, the spatial migration range of the CO_2_ fluid gradually expands with increasing storage time, although the spatial distribution pattern of gas-phase CO_2_ does not experience substantial changes. Due to the increased dissolution of CO_2_, the CO_2_-specific gravity (SG) in the center of the injection well area decreases rapidly, from the initial values of 0.9 and 0.95 to 0.28, 0.48, 0.4, and 0.55 at 1000 years, respectively. The diffusion range of the CO_2_ plume increases over time, and by 1000 years, the leading edge of the CO_2_ plume in all four reservoirs is within 4 km in the horizontal direction, which is within the safety evaluation range. The lateral spread distances of the CO_2_ plume in the four reservoirs are 4 km, 3 km, 3.6 km, and 3.1 km, respectively (Fig 8).

In the early stages of injection, CO_2_ in all four reservoirs is primarily in the free phase, followed by the bound phase and dissolved phase. Except for the free phase CO_2_, the storage capacity of CO_2_ in the other phases gradually increases over time. As the proportion of free-phase CO_2_ decreases, the global proportion of residual and dissolved CO_2_ continues to rise, with the dissolved-phase storage increasing significantly over time. After 500 years, CO_2_ mineralization begins to show its effects, and the transformation of CO_2_ in deep saline aquifers through different mechanisms becomes a key factor influencing the storage safety of deep saline formations. The extent of transformation of supercritical CO_2_ into dissolved and mineralized forms is an important factor for evaluating the storage capacity of deep saline aquifers. By the 1000-year mark, the evolution of all four storage mechanisms becomes more apparent in the four reservoirs. The mass percentage of gas-phase CO_2_ in each reservoir decreases from 88.7%, 85.1%, 87.7%, and 85.4% at the time injection stops to 58.4%, 66.9%, 53.0%, and 58.6% at 1000 years. The amount of free-phase CO_2_ significantly decreases, while the storage in the other three mechanisms gradually increases. CO_2_ dissolves into the saline water in the reservoirs, and over time, the CO_2_ content dissolved in the target saline aquifers gradually increases. Since the initiation of CO_2_ injection, the CO_2_ dissolved in the saline water near the wellbore gradually increases and becomes saturated. Meanwhile, CO_2_ continues to dissolve through radial and vertical flow, causing large amounts of supercritical CO_2_ to interact with the formation water. Due to the higher density of saline water containing large amounts of dissolved CO_2_, this portion of the saline water moves downward. In contrast, saline water with less or undissolved CO_2_, having a lower density, moves upward. Under the influence of pressure gradients, the dissolution of CO_2_ in the saline aquifer increases, leading to more dissolved CO_2_ in the formation water. At the 1000-year mark, the content of dissolved CO_2_ in each storage layer accounts for 37.6%, 28.6%, 38.7%, and 37.38% of the total, respectively(Table 4).

**Table 4:** Percentage of sequestration quality by different sequestration mechanisms in various reservoirs at different time intervals.

ReservoirTime		5a	10a	50a	100a	500a	1000a
S2 Reservoir	Free Phase	88.40%	88.71%	77.80%	71.68%	55.43%	45.88%
	Dissolved Phase	11.58%	11.20%	19.80%	23.60%	35.70%	37.60%
	Residual Phase	0.02%	0.005%	2.30%	3.50%	7.20%	12.50%
	Mineralized Phase	0.0003%	0.08%	0.097%	1.22%	1.67%	4.02%
S3-1Upper Reservoir	Free Phase	83.88%	84.97%	82.26%	72.77%	59.72%	53.72%
	Dissolved Phase	15.70%	14.2%	17.20%	19.20%	28.60%	28.60%
	Residual Phase	0.23%	0.21%	0.06%	5.95%	8.40%	13.20%
	Mineralized Phase	0.19%	0.65%	0.48%	2.08%	3.28%	4.48%
S3-1Lower Reservoir	Free Phase	86.37%	87.4%	82.04%	65.30%	55.31%	34.28%
	Dissolved Phase	13.50%	12.17%	16.50%	28.90%	34.20%	38.70%
	Residual Phase	0.06%	0.03%	1.14%	4.28%	6.97%	18.70%
	Mineralized Phase	0.07%	0.40%	0.32%	1.52%	3.52%	8.32%
S3-2 Reservoir	Free Phase	83.70%	84.60%	70.90%	64.80%	49.00%	45.70%
	Dissolved Phase	15.70%	14.31%	26.66%	28.82%	36.68%	37.38%
	Residual Phase	0.25%	0.82%	2.16%	5.15%	11.50%	12.90%
	Mineralized Phase	0.27%	0.27%	0.28%	1.23%	2.82%	4.02%

## Discussion

In this paper, an evaluation of CO2 geological storage sites in the Yellow River Delta region is conducted. Through the establishment of an evaluation index system, GIS - based spatial processing of multivariate information, and numerical simulation of CO2 geological storage in the selected target areas, certain results have been obtained. However, there are the following issues that require continuous research and improvement:

(1) The evaluation of CO2 geological storage sites is a dynamic process throughout the entire life cycle of CO2 geological storage. This paper covers data preparation and analysis, field visits and investigations, stratigraphic surveys and explorations, as well as model inferences. Nevertheless, it lacks research on leakage monitoring after injection. Therefore, in future studies, a comprehensive evaluation of the storage sites should be carried out to enhance injection efficiency, economic viability, and safety.
(2) When establishing the evaluation index system for CO2 geological storage sites, it is necessary to comprehensively consider influencing factors and select appropriate evaluation indices. The evaluation and screening of target sites involve the mutual coupling of multiple factors. Currently, due to the limited understanding of the study area and the immaturity of related geological background research, the CO2 storage site evaluation index system established in this paper needs further refinement. Additionally, as relevant research in China started relatively late, the relevant laws, regulations, and construction standards are not yet fully developed, which restricts the selection of relevant evaluation indices. With the continuous improvement of relevant laws, regulations, and engineering construction standards, the evaluation index system should be further optimized in future research to increase the accuracy of evaluation results.
(3) In this paper, when conducting a comprehensive analysis of multivariate information based on GIS, some influencing factors cannot be directly processed spatially with other factors, and human - computer interaction is employed, which affects the evaluation results. In the future, a scientific approach should be adopted to establish and clean the multivariate spatial information dataset. Moreover, although this paper has completed the numerical simulation of CO2 geological storage in the target area, in the simulation of the grid section during numerical simulation, due to the complexity of the geological body, the grid section fails to fully and accurately simulate the actual situation. In future research, further exploration is needed to develop an integrated method that can achieve a double - optimal balance between the grid section and data volume, enabling accurate simulation of all aspects of CO2 geological storage characteristics.

## Conclusion

This study establishes an evaluation index system for CO₂ geological sequestration sites in the Yellow River Delta, integrating GIS spatial analysis to delineate suitability zones and target reservoirs by comprehensively assessing factors such as drinking water sources, ecologically sensitive areas, and protected regions. The analysis reveals four high-density aggregation zones of CO₂ emission sources within the study area. By applying saline aquifer sequestration principles and GIS proximity analysis, optimal source-reservoir matching was achieved under a 100 km search radius, enabling full coverage of emission sources and sequestration targets. Numerical simulations demonstrate that pressure variations in the four sequestration layers remained within safe thresholds during and after CO₂ injection. The migration patterns exhibited consistent characteristics: a funnel-shaped distribution during injection, buoyancy-driven upward migration post-injection with capillary breakthrough, followed by lateral diffusion. By the simulation’s conclusion, lateral CO₂ migration in all layers remained confined within predefined safety boundaries. Notably, the deep saline aquifer of the Shahejie Formation in the target area demonstrated superior CO₂ injectivity, storage capacity, and minimal sequestration risks, providing robust scientific support for the implementation of regional CO₂ geological sequestration projects.

## Supporting information

S1 File Numerical simulation. (ZIP)
